# Supplementary material for: Causal Relationship between Aging and Anorexia Nervosa: A White-Matter-Microstructure-Mediated Mendelian Randomization Analysis
Source: Biomedicines. 2024 Aug 16;12(8):1874. doi: 10.3390/biomedicines12081874 (PMC11351342; doi:10.3390/biomedicines12081874)
Supplement: Supplementary file 1 [file biomedicines-12-01874-s001.zip › Supplementary Figure/Figure S4/Singe SNP plot of MR analysis for TL to ubm-b-1601.pdf]

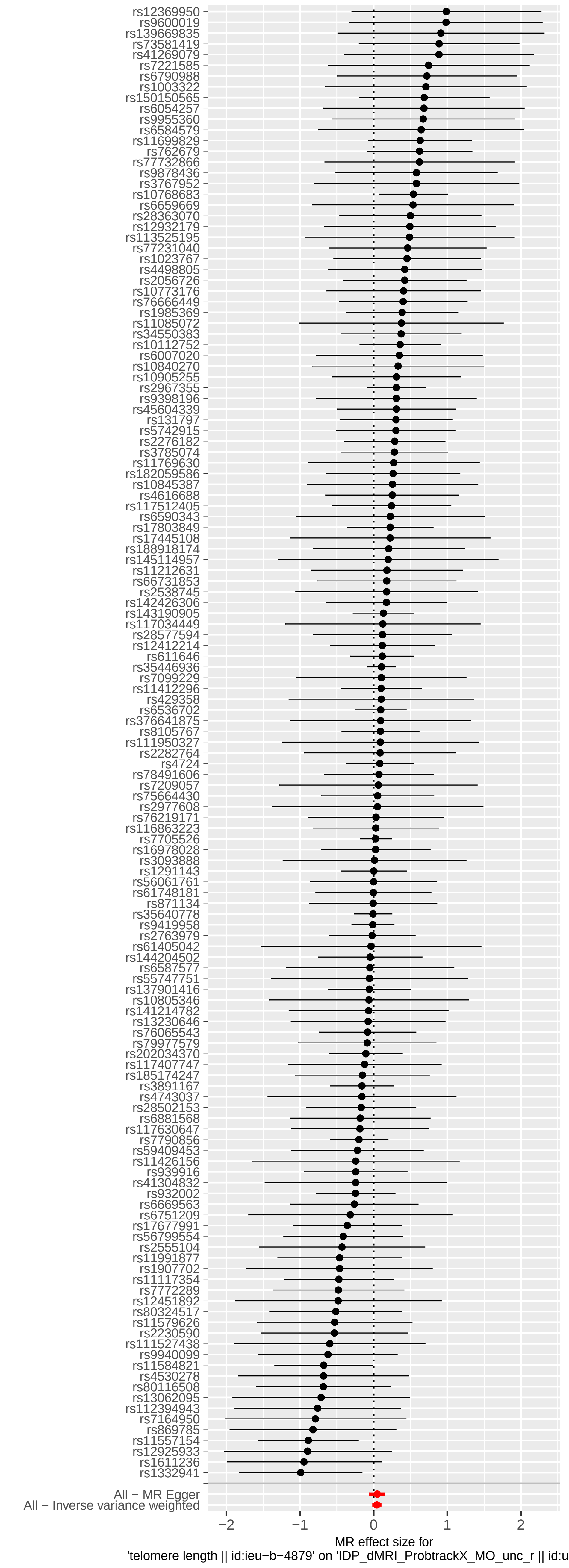
rs12369950
rs9600019
rs139669835
rs73581419
rs41269079
rs7221585
rs6790988
rs1003322
rs150150565
rs6054257
rs9955360
rs6584579
rs11699829
rs762679
rs77732866
rs9878436
rs3767952
rs10768683
rs6659669
rs28363070
rs12932179
rs113525195
rs77231040
rs1023767
rs4498805
rs2056726
rs10773176
rs76666449
rs1985369
rs11085072
rs34550383
rs10112752
rs6007020
rs10840270
rs10905255
rs2967355
rs9398196
rs45604339
rs131797
rs5742915
rs2276182
rs3785074
rs11769630
rs182059586
rs10845387
rs4616688
rs117512405
rs6590343
rs17803849
rs17445108
rs188918174
rs145114957
rs11212631
rs66731853
rs2538745
rs142426306
rs143190905
rs117034449
rs28577594
rs12412214
rs611646
rs35446936
rs7099229
rs11412296
rs429358
rs6536702
rs376641875
rs8105767
rs111950327
rs2282764
rs4724
rs78491606
rs7209057
rs75664430
rs2977608
rs76219171
rs116863223
rs7705526
rs16978028
rs3093888
rs1291143
rs56061761
rs61748181
rs871134
rs35640778
rs9419958
rs2763979
rs61405042
rs144204502
rs6587577
rs55747751
rs137901416
rs10805346
rs141214782
rs13230646
rs76065543
rs79977579
rs202034370
rs117407747
rs185174247
rs3891167
rs4743037
rs28502153
rs6881568
rs117630647
rs7790856
rs59409453
rs11426156
rs939916
rs41304832
rs932002
rs6669563
rs6751209
rs17677991
rs56799554
rs2555104
rs11991877
rs1907702
rs11117354
rs7772289
rs12451892
rs80324517
rs11579626
rs2230590
rs111527438
rs9940099
rs11584821
rs4530278
rs80116508
rs13062095
rs112394943
rs7164950
rs869785
rs11557154
rs12925933
rs1611236
rs1332941
All − MR Egger
All − Inverse variance weighted
−2
−1
0
1
2
MR effect size for
'telomere length || id:ieu−b−4879' on 'IDP_dMRI_ProbtrackX_MO_unc_r || id:u
